# A Fine-Tuned Multimodal AI Chatbot for Dietary Health and Nutrition, Purrfessor: Development and Mixed Methods Evaluation

**DOI:** 10.2196/74111

**Published:** 2026-04-30

**Authors:** Linqi Lu, Yifan Deng, Chuan Tian, Sijia Yang, Dhavan V Shah

**Affiliations:** 1 Department of Communication, University of North Dakota, 221 Centennial Drive, Stop 7169, Grand Forks, ND, 58202, United States, 1 7017772137; 2 Department of Computer Science, University of Wisconsin–Madison, Madison, WI, United States; 3 School of Journalism and Mass Communication, University of Wisconsin–Madison, Madison, WI, United States

**Keywords:** artificial intelligence, Large Language and Vision Assistant, LLaVA, computer vision, large language models, LLMs, digital health, diet health, health technology, nutrition, diet recommendation, chatbot

## Abstract

**Background:**

The integration of Large Language and Vision Assistant models with food and nutrition data enables multimodal meal analysis and contextual dietary guidance. Despite this potential, the reliability and practical usefulness of such systems for supporting everyday dietary decision-making remain underexplored.

**Objective:**

This study introduces Purrfessor, an innovative artificial intelligence (AI) chatbot designed to provide personalized dietary guidance through interactive, multimodal engagement. The study aimed to evaluate its performance in ingredient recognition and recipe generation.

**Methods:**

The Purrfessor chatbot was trained using a combination of the FoodData Central database from the US Department of Agriculture (USDA), the Recipe2img dataset featuring food images and corresponding recipes, a curated human-annotated dataset derived from Recipe1M, and a customized question-and-answer dialogue dataset. The system operates under a session-based, multiturn interaction paradigm, with memory retained only within an active session and no cross-session memory persistence. We implemented a 2-phase evaluation framework combining AI-based performance assessment and human scoring.

**Results:**

Purrfessor achieved a high average cosine similarity of 0.90 in ingredient recognition with human-coded references. In GPT-4.1–based (OpenAI) evaluation of recipe generation quality, Purrfessor outperformed the raw Large Language and Vision Assistant model across all evaluated dimensions, with the largest improvements in completeness (7.44 vs 6.52), consistency (8.90 vs 7.81), and clarity (9.13 vs 8.39). Overall recipe quality improved from 7.66 to 8.35. Automatic metrics indicated strong ingredient coverage (0.78) and moderate step complexity (0.74), with lower coherence (0.62) and temperature and time specification (0.59), yielding an overall structured score of 0.68. Human evaluators rated Purrfessor’s question-and-answer accuracy highly: correctness (mean 8.71, SD 1.15), relevance (mean 9.99, SD 0.10), and clarity (mean 9.33, SD 0.68). Error analysis indicated that 56% of responses contained minor hallucinations (ie, inclusion of inferred secondary details or invisible garnishes). At the same time, core food identification and overall recipe logic remained accurate.

**Conclusions:**

Findings highlight the role of anthropomorphic chatbot design and multimodal AI in supporting engaging dietary health conversations. This study offers an example of AI-driven, evidence-based dietary guidance and underscores the potential of health chatbots to nudge informed health decision-making. Insights contribute to the development of digital health interventions and personalized health communication strategies, with implications for the design of engaging, user-centered AI health assistants.

## Introduction

### Overview

Artificial intelligence (AI) chatbots have increasingly become integrated into daily human interactions, assisting users across a range of domains, from customer service to personal health management. Despite the widespread potential, individuals in underserved communities frequently encounter significant barriers to healthy meal planning, including limited grocery options, economic constraints, and time pressures [1-3]. The evolution from rule-based systems to advanced chatbots that leverage natural language processing, machine learning, and large language models (LLMs) has enabled real-time, context-sensitive interactions tailored to individual behavior. AI-powered conversational agents are transforming health care and lifestyle management, particularly in health interventions. Unlike traditional health-tracking apps (eg, MyFitnessPal; MyFitnessPal, Inc), AI-enhanced chatbots provide dynamic, adaptive guidance, fostering personalized user engagement [4,5]. AI-driven health chatbots hold significant promise in addressing key behavioral factors associated with chronic conditions, including cardiovascular disease, type 2 diabetes, and obesity [6,7]. While mainstream health apps offer static tracking, they lack the interactive and adaptive capabilities of AI chatbots, which integrate multimodal inputs for more responsive and personalized health guidance [8,9]. Leveraging computer vision in conjunction with LLMs, chatbots can analyze user-uploaded meal images, deliver prompt dietary feedback, and enhance their role in health behavior modification [10,11].

This study introduces Purrfessor, a multimodal chatbot designed to provide personalized dietary guidance by fine-tuning the LLaVA-v1.6‐13B base model to enable advanced interactive functionalities [12]. By leveraging user-uploaded food images and text prompts, Purrfessor uses structured data to map food types to nutritional information and recipe suggestions, enhancing the accuracy and relevance of dietary assessments [11,13]. This study explores how AI-driven chatbots can serve as engaging health companions [10,14]. By providing prompt, accessible meal suggestions based on available ingredients, the chatbot aims to help individuals and families build nutrition knowledge and support healthier dietary habits [1,15,16]. This research contributes to the development of AI-driven health assistants that move beyond static recommendations, offering dynamic, context-aware support for sustained lifestyle modifications [9,17].

### Advancements in Multimodal AI for Health Chatbots

The integration of multimodal AI has expanded the capabilities of health chatbots, enabling dynamic, personalized dietary interventions. Unlike traditional health-tracking apps that rely on static databases, AI-driven chatbots can process real-time user inputs, including text and images, to offer adaptive health recommendations [4,11]. Studies indicate that chatbots can improve adherence to health guidelines and promote sustained behavior change [9,18]. However, limitations remain, particularly in terms of accuracy, engagement, and trustworthiness. AI-generated recommendations risk hallucinations (fabricated content), inconsistent advice, and privacy concerns, posing challenges for dietary-tracking apps [3,19,20]. Addressing these challenges is crucial to enhancing user trust and long-term engagement in AI-powered health interventions [1,2].

Recent advancements in multimodal AI have sought to improve chatbot accuracy and engagement, with instruction tuning emerging as a key technique in refining AI-driven interactions. Large Language and Vision Assistant (LLaVA) represents one such innovation, integrating a visual encoder with an LLM to process combined image-text inputs. By leveraging structured instruction tuning, LLaVA enhances the chatbot’s ability to interpret visual dietary data and generate relevant feedback, aligning closely with state-of-the-art language models and achieving more than 85% of GPT-4’s (OpenAI) accuracy in image-text reasoning tasks [12]. This development supports applications where AI-powered chatbots analyze user-uploaded images of meals, providing prompt, tailored dietary assessments [11].

### Anthropomorphism and Chatbot Image

Chatbots that exhibit social characteristics tend to foster stronger relational bonds with users, thereby enhancing user engagement and adherence to health [9]. However, delivering health interventions can be challenging because individuals often resist advice perceived as threatening their established habits or personal autonomy [21]. Such resistance typically arises from psychological reactance, in which users perceive health advice as restrictive or authoritative, leading to disengagement or counterarguments [22].

Anthropomorphism, the attribution of human-like qualities to nonhuman entities, offers an effective strategy to mitigate this resistance. By displaying relatable, human-like traits, chatbots can facilitate more natural and socially engaging interactions [23]. Attachment theory further supports this approach, suggesting users are more likely to develop emotional connections, trust, and ultimately adhere to health recommendations when interacting with chatbots that possess relatable, personable characteristics [24]. Moreover, integrating interpersonal communication theories into chatbot interactions can enhance relational dynamics, thereby improving their capacity to influence user behavior [25].

According to the Media Are Social Actors paradigm, users respond socially to media entities that display recognizable social cues, even when they acknowledge these entities as artificial constructs [26]. Adopting an approachable, cat-themed professor persona is a potential way to leverage these social interaction principles. This playful and companionable persona not only reduces resistance by avoiding an authoritative tone but also fosters an enjoyable, collaborative atmosphere that motivates users to explore dietary health behaviors [27].

## Methods

### System Overview

**Figure 1.**
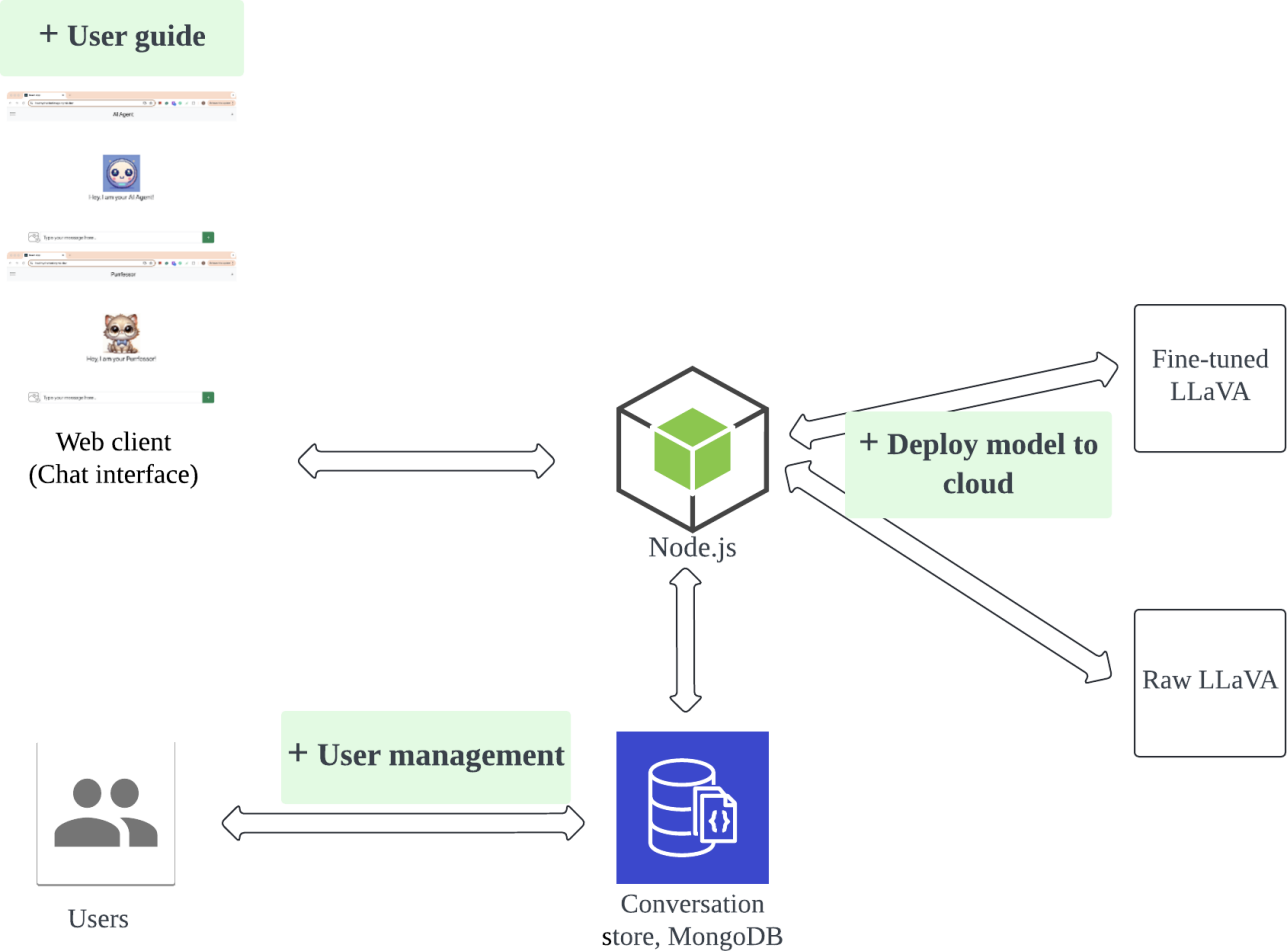
System architecture. LLaVA: Large Language and Vision Assistant.

The Purrfessor chatbot is an AI-powered dietary assistant integrating natural language processing and computer vision to provide personalized nutrition guidance. The system architecture consists of a web-based user interface, a backend server facilitating data exchange, a conversation database for storing user interactions, and cloud-hosted AI models for text and image processing (Figure 1). The workflow enables near real-time meal analysis, dietary recommendations, and interactive chatbot engagement.

### System Components

The user interaction module provides a web-based chat interface for text-based conversations or for uploading food images for dietary analysis. The chatbot is visually represented by a cat-themed persona, “Purrfessor,” designed to convey an approachable yet knowledgeable character and enhance user engagement. The system also supports user authentication, enabling personalized dietary recommendations based on stored preferences and meal history. However, in the evaluated deployment, interactions are processed in a session-based setting without the retrieval of conversational history across sessions.

The backend infrastructure is implemented using a Node.js server that facilitates communication among the user interface, AI models, and database services. The server manages HTTP requests, processes application programming interface (API) calls, and maintains session continuity. User interactions are stored in a MongoDB conversation database, enabling conversational persistence, analysis of engagement patterns, and incremental personalization of responses over time.

The fine-tuned LLaVA model is deployed in a cloud-based computing environment to support scalable inference and multimodal processing. Cloud hosting enables efficient handling of image inputs and response generation for multiple concurrent users, while also supporting model updates and iterative fine-tuning to improve response accuracy and system performance.

### User Interaction and Engagement Features

**Figure 2.**
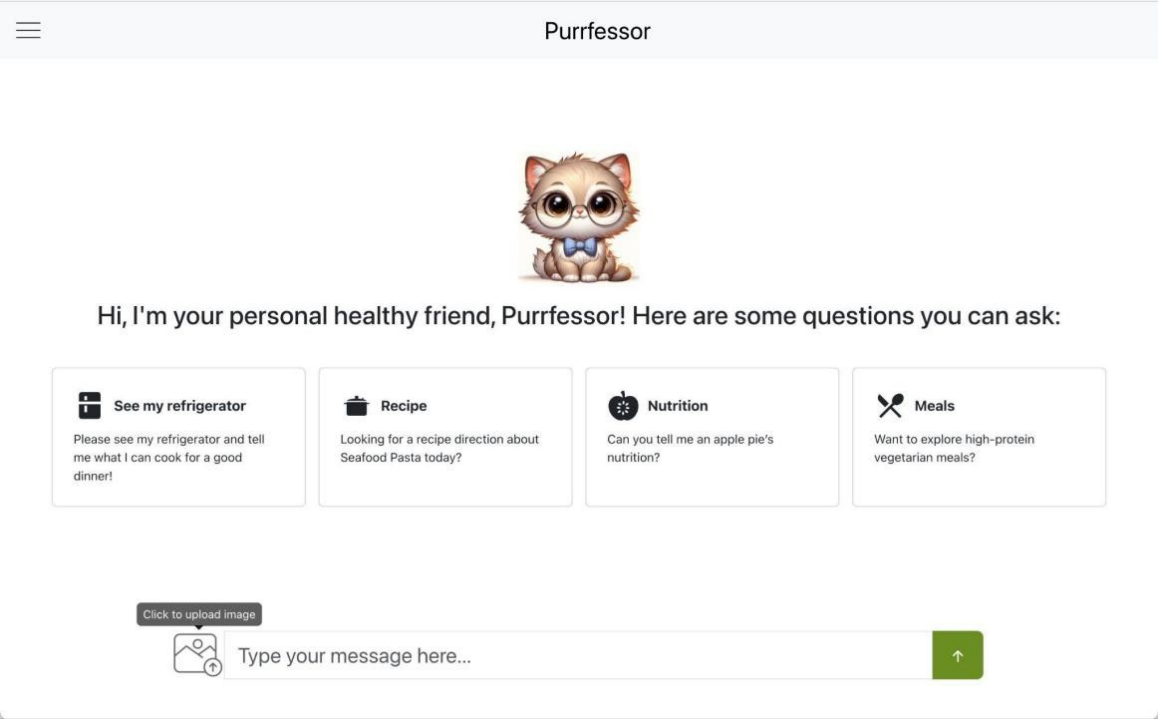
Purrfessor interface [28].

The chatbot interface (Figure 2) is designed to support intuitive, seamless user interaction through visual prompts and guided navigation. To facilitate onboarding and early exploration, the interface displays prompt suggestions above the conversation bar that illustrate common interaction scenarios, such as requesting recipe ideas based on refrigerator contents or exploring specific dietary preferences. These prompts can be selected to automatically populate the input field, reducing user effort and lowering the barrier to initial engagement.

Navigation support is further enhanced by menu icons supplemented with tooltip-based hints that provide brief, context-specific explanations when users hover over or tap an icon (eg, “Click here to generate a custom meal plan based on your preferences”). Additional assistance is available via “?” icons adjacent to advanced features, allowing users to access clarification without disrupting the interaction flow. The interface also includes a visually distinct image upload button that enables users to submit photos of meals or ingredients for AI-driven analysis. Visual cues, such as color changes, encourage image uploads and highlight the chatbot’s multimodal capabilities. For first-time users, a guided introduction outlines key functionalities, including prompt selection, image uploads, and navigation, demonstrating how to initiate conversations using example prompts or custom questions and reducing initial uncertainty during early use.

### Response Latency Metrics

We report response latency metrics for the system configuration used in all evaluations presented in this study (2025 deployment). The system uses streaming inference with a virtual large language model on a single NVIDIA RTX 6000 Ada Graphics Processing Unit (GPU). For a typical response of approximately 500 tokens, input token processing required approximately 2‐3 seconds, after which output tokens were streamed to the user as they were generated. Full response generation, including server-side processing and database forwarding, was completed within approximately 10‐12 seconds.

### Training Data Sources

The fine-tuning dataset was curated from three sources: (1) FoodData Central: Foundation Foods, which is a structured food and nutrition database from the US Department of Agriculture (USDA), which provides detailed nutritional profiles [29]; (2) Recipe2img dataset: a dataset containing paired food images and recipe descriptions, designed for learning cross-modal representations between text and visuals [11]; and (3) a human-annotated dataset: a curated collection of food images labeled with nutritional information and cooking instructions, derived from the Recipe1M dataset. This dataset was used to construct instruction-tuning prompts and to enhance the contextual relevance of dietary recommendations.

### Human-Annotated Data Preparation

First, a Google Image Search method was implemented to systematically collect food-related images from online sources using Python (Python Software Foundation) and the Google Custom Search API, an approach adapted from similar image-retrieval practices in data-intensive research [30,31]. The collection process was facilitated through the Google API Python Client library [32], with a custom search engine ID and API key configured to automate retrieval. A set of predefined search queries, tailored with keywords on raw produce and ingredients for cooking (ie, “raw food,” “produce and meat in fridge,” “fresh produce,” “produce in fridge,” “food ingredients,” “raw meat and produce,” “cooking raw meat and vegetables,” and “fresh produce for cooking”) was used to filter relevant images and metadata. Metadata extracted for each image included the collection date, page title, source website, image URL, and page URL for image validation use. Where available, dates were extracted directly from URLs using regular expressions, allowing chronological organization of data [33]. Images were accessed via licensed use of Google Custom Search API in compliance with Google’s terms of service.

Second, the image captioning and corresponding question-and-answer (Q&A) were generated via GPT-4o (OpenAI). Each image in the dataset was processed using a prompt specifically designed to elicit detailed captions and chatbot-friendly Q&A examples. The prompt instructed the model to generate the following: a detailed caption describing the visible ingredients; a Q&A response including a greeting, nutritional information for the ingredients, healthy recipe suggestions, a step-by-step guide for each recipe, and a closing message encouraging user engagement. To maintain consistency, each prompt followed a structured output format, facilitating easy parsing and subsequent training of the LLaVA model. The inclusion of nutritional information and recipe suggestions was essential to simulate real-world interaction, thereby training the model to respond informatively and engagingly [34].

Third, we completed the human review and editing for the compiled GPT (OpenAI) output. Following the initial output generation, human annotators reviewed each caption and Q&A example for clarity, accuracy, and alignment with expected chatbot responses. Annotators checked for language appropriateness, grammatical correctness, logical flow, edge case handling, and special characters. For ambiguous or nonfood images, annotators crafted captions and Q&A responses neutrally, providing factual descriptions of the content per the prompt guidelines [33]. Additionally, edge cases were included in the full dataset, with Q&A examples revised by humans, ensuring robust training data that would prepare the model to handle diverse real-world scenarios.

### Chatbot Fine-Tuning Approach

The chatbot was fine-tuned using LLaVA-v1.6-Vicuna-13B, a multimodal LLM that integrates an open-set visual encoder from Contrastive Language–Image Pretraining with the Vicuna language decoder. Fine-tuning was performed on a high-performance GPU computing server featuring dual NVIDIA RTX 6000 Ada Generation GPUs (48 GB VRAM per GPU; 18,176 CUDA cores; and 568 Tensor cores), a 64-core CPU, and 512 GB RAM. Training used Ubuntu 22.04 with the Lambda Stack environment. The designed architecture of Purrfessor enables the model to process and interpret both visual and textual inputs within instruction-based contexts, making it suitable for tasks such as image interpretation, dialogue reasoning, and dietary guidance [12]. The data structure contains (1) the Food Data Central: Foundation Foods from the USDA, (2) Recipe2img dataset Salvador et al [11] (n=3000), and (3) a human-annotated visual dataset (n=500). The instruction-tuning dataset also included supportive opening and closing language, enhancing the emotional support provided to users.

Initially, full fine-tuning of LLaVA-v1.6-Vicuna-13B was attempted using the Recipe1M dataset. However, the high GPU memory requirements for tuning a 13B parameter model exceeded hardware constraints. To address this, Low-Rank Adaptation (LoRA) was implemented, allowing efficient fine-tuning by modifying a subset of model parameters. Early LoRA experiments revealed overfitting, where the model defaulted to generating generic cooking instructions for all queries, irrespective of context. To mitigate this issue, additional human-annotated visual data were introduced to improve context-awareness in meal recommendations. Fine-tuning incorporated instruction-tuning prompts that emphasized ingredient recognition and personalized dietary suggestions rather than generic recipe generation. To ensure the chatbot-generated precise nutrition-related responses, the Central Foundation Foods dataset was integrated into the instruction tuning. Knowledge injection techniques refined the chatbot’s ability to distinguish food components and provide relevant nutritional insights. Additionally, structured prompt engineering ensured that chatbot interactions maintained a natural conversational flow, balancing factual dietary information with engaging, supportive dialogue.

The fine-tuned Purrfessor chatbot integrates AI-driven dietary recommendations with personalized, image-based analysis. The model’s training pipeline optimized its ability to deliver context-aware meal-planning suggestions, reducing bias toward predefined responses and improving its adaptability to user-specific queries.

### Evaluation Framework

To assess the performance of the fine-tuned visual chatbot Purrfessor, we adopted a mixed methods, 2-phase evaluation framework. This design integrated both automated scoring mechanisms and human validation procedures to capture technical performance, contextual relevance, semantic robustness, and user-centered feedback.

### Simulation Dataset

This study randomly selected 100 real-world images from a second-round Google Image Search pool, which served as a held-out evaluation set and did not overlap with any training or fine-tuning data. To emulate authentic use cases, we paired each image with a synthetically generated user prompt, yielding 100 Q&A pairs. Image content represented a wide range of food types, including everyday meals, beverages, and raw ingredients, to reflect the variety users might encounter in real-world applications. Prompt diversity included direct identification tasks (eg, “What food-related items appeared in this image?”) and contextual queries (eg, “Do you have ideas on recipes with low-fat per the current ingredients I provide?”). This ensured the 100 Q&A pairs captured both fundamental visual object detection and high-level reasoning in meal planning. To evaluate the food detection and recipe quality, prompts were designed to reflect a range of interaction intents, including visual identification tasks (eg, “Please comprehensively list all food-related items appeared in this image?” and “Please only list food-related items appeared in this image with high confidence”), and general recipe generation (eg, “Based on this image, what food recipe would you recommend?”).

### Food Detection Performance

To assess ingredient recognition capabilities, we implemented a semantic evaluation pipeline based on Bidirectional Encoder Representations from Transformers, using the all-MiniLM-L6-v2 model. Ground truth ingredient labels were human-coded, and model predictions were compared using a cosine similarity-based matching algorithm with a minimum semantic threshold of 0.7. Evaluation was performed under 3 prompt conditions: comprehensive detection (the model was prompted to list all visible food items exhaustively), confident detection (the model was prompted to name only items it could identify with high confidence), and naturalistic prompting (the model was given open-ended prompts such as, “What food recipe would you recommend based on this image?” without explicitly requesting full ingredient enumeration).

### Recipe Quality: LLM-as-a-Judge

To evaluate recipe generation, we adopted the G-Eval framework [35], a chain-of-thought evaluation method implemented using GPT-4.1 as an expert synthetic evaluator. Each generated recipe was evaluated across 7 key dimensions: correctness (alignment with image content), relevance (task adherence), clarity (readability and structure), completeness (coverage of cooking steps), consistency (logical flow), practicality (procedural feasibility of executing the recipe once the required ingredients are available), and safety (food safety awareness). Scores were assigned on a 10-point Likert scale, and a weighted average was computed as the overall quality metric. This “LLM-as-a-Judge” method has been increasingly validated for evaluating natural language generation systems. Each recipe sample was evaluated based on its alignment with the corresponding food image and user prompt. The evaluation script passed three structured components to the model: (1) the user prompt, (2) the generated recipe, and (3) the base64-encoded image. A system prompt instructed GPT-4.1 to follow a stepwise chain-of-thought procedure involving (1) visual analysis of the dish image, (2) breakdown of the recipe content (eg, ingredients, steps, and safety), and (3) cross-modal validation between image and text.

### Recipe-Specific Automatic Evaluation

To complement LLM-based evaluation, we applied a reference-free, domain-specific scoring framework [36]. Four structured metrics were implemented: ingredient coverage (consistency between ingredients listed in the “Ingredients Overview” and those used in the recipe body), step complexity (capturing instructional richness through operation diversity, sentence length, and parameter inclusion), recipe coherence (validating logical sequencing and completeness using rule-based checks), and temperature and time specification (the presence and plausibility of safety-critical parameters). Scores ranged from 0 to 1. An overall weighted quality score was computed using the following weights: coverage (0.3), complexity (0.2), coherence (0.3), and specification (0.2). The system is implemented in Python 3.8 (Python Software Foundation) using *NLTK* and *NumPy* and supports scalable recipe evaluation.

### Human Evaluation

Three human coders manually reviewed and rated each of the 100 chatbot-generated responses based on correctness, relevance, and clarity (MultimediaAppendices 1  2). Two raters were independent research assistants who volunteered their time, and one rater was a study author. All raters received the same training and applied identical evaluation criteria. Each dimension was rated on a 10-point scale, adapted from validated chatbot assessment frameworks [16]. Coders were trained before evaluation, and a scoring rubric was provided to ensure interrater consistency. Krippendorff α was calculated to assess intercoder reliability, which ranged from 0.85 to 1.00 across dimensions.

### Ethical Considerations

This study used a simulation-based evaluation design and did not involve direct interaction with human participants for research data collection. Human evaluation was conducted on chatbot-generated responses rather than on human behavioral or personal data. No personal or sensitive data were collected or analyzed. As no human participants were recruited, informed consent was not required, and no compensation was provided. All electronic research data were stored on secure, password-protected institutional servers accessible only to authorized research team members. The system evaluated in this study operates under a session-based configuration without cross-session memory persistence, and no real user interaction data were collected or analyzed for this research. This research project was reviewed and approved by the Institutional Review Board at the University of Wisconsin–Madison (2023‐1416).

## Results

### Food Detection Evaluation

We first evaluated ingredient recognition performance across 3 prompting scenarios. In the comprehensive detection task, where the chatbot was instructed to identify all visible ingredients exhaustively, the average cosine similarity between predicted and ground-truth ingredient pairs improved from 0.80 with the original raw LLaVA model to 0.84 with the fine-tuned Purrfessor model, reflecting a 5% gain in semantic alignment. In the confident prompt condition, where the chatbot was asked to list only clearly visible ingredients, similarity further increased from 0.85 to 0.87 (+2%).

To assess model behavior under naturalistic prompting conditions, we conducted a simulation using 100 randomly sampled, image-based user queries (eg, “Based on the image, what food recipe would you recommend?”). Although exhaustive ingredient enumeration was not explicitly requested, chatbot responses consistently included an “Ingredient Overview” section listing salient food items. When benchmarked against human-coded labels, the model achieved a precision of 0.65, indicating that the majority of predicted ingredients were relevant. The average cosine similarity of 0.90 across matched ingredient pairs suggests strong semantic alignment, even in the absence of exact lexical matches.

### Recipe Quality Evaluation

To compare end-to-end recipe generation quality across models, we used the LLM-as-a-Judge framework with GPT-4.1 in the G-Eval structured evaluation pipeline [35]. For each sample, models were prompted with “Using the food and ingredients shown in this image, please recommend a healthy recipe and estimate its nutritional information.” Generated outputs were rated across 7 dimensions to yield an overall quality score [35]: correctness, relevance, clarity, completeness, consistency, practicality, and safety, using a 10-point scale. A weighted average was computed to produce an overall quality score. As shown in Table 1, the Purrfessor model consistently outperformed the raw LLaVA model across all 7 dimensions. Paired *t* tests were conducted on 100 matched samples to compare the fine-tuned Purrfessor model with the raw LLaVA baseline. Results indicated that the fine-tuned model achieved significantly higher scores across all 7 evaluation dimensions, including correctness (*t*_99_=4.23; *P*<.001), relevance (*t*_99_=3.11; *P*=.002), clarity (*t*_99_=6.03; *P*<.001), completeness (*t*_99_=6.95; *P*<.001), consistency (*t*_99_=7.45; *P*<.001), practicality (*t*_99_=4.28; *P*<.001), and safety (*t*_99_=5.10; *P*<.001).

**Table 1.** GPT-4.1 (G-Eval) structured evaluation of recipe generation quality comparing the raw LLaVA-v1.6-13B model and the fine-tuned Purrfessor multimodal dietary chatbot across 100 simulated food image–prompt pairs (United States, 2025 system deployment).^a^

Evaluation dimension	Raw LLaVA^b^, mean (SD)	Purrfessor, mean (SD)	Absolute improvement	*P* value
Correctness	6.14 (1.53)	6.72 (1.52)	0.58	<.001
Relevance	8.01 (1.73)	8.47 (1.79)	0.46	.002
Clarity	8.39 (1.18)	9.13 (0.87)	0.74	<.001
Completeness	6.52 (1.32)	7.44 (1.06)	0.92	<.001
Consistency	7.81 (1.45)	8.90 (1.11)	1.09	<.001
Practicality	8.52 (1.27)	9.11 (1.12)	0.59	<.001
Safety	8.24 (1.27)	8.67 (1.02)	0.43	<.001
Overall score (weighted average)	7.66	8.35	0.69	—^c^

a Recipes were generated in response to image-based dietary guidance queries and evaluated using a large language model-as-a-judge framework (GPT-4.1) across 7 dimensions: correctness, relevance, clarity, completeness, consistency, practicality, and safety (10-point Likert scale). Paired *t* tests were conducted on 100 matched samples to assess improvements attributable to domain-specific fine-tuning. The overall weighted score represents a composite across all 7 dimensions. All evaluations were conducted under a session-based deployment configuration without cross-session memory persistence.

b LLaVA: Large Language and Vision Assistant.

c Not applicable.

To complement human-based assessment, we implemented a domain-specific automatic evaluation framework based on four reference-free, structure-aware metrics: (1) ingredient coverage, (2) step complexity, (3) recipe coherence, and (4) temperature and time specification. These metrics emphasize internal consistency and executability, independent of reference text similarity. On the same 100 image-prompted samples, the model achieved an ingredient coverage score of 0.78, reflecting strong alignment between the “Ingredients Overview” and ingredients actually used in recipe steps. The step complexity score of 0.74 indicated moderately detailed instruction sets with diverse cooking operations. Recipe coherence was lower at 0.62, revealing some violations of expected temporal and logical sequencing (eg, adding ingredients before preheating). The temperature and time specification score of 0.59 suggested partial inclusion of cooking duration and temperature parameters. The aggregated overall structured evaluation score was 0.68, placing recipe quality in the good range, with opportunities for improvement in procedural coherence and parameter completeness.

### Human Evaluations

Human evaluation was conducted on 100 Q&A simulations generated by Purrfessor, assessing 3 core criteria (Table 2).

**Table 2.** Human evaluation of 100 simulated image-based dietary Q&A responses generated by the fine-tuned Purrfessor multimodal chatbot (United States, 2025 system deployment).^a^

Evaluation criteria	Mean (SD)	Krippendorff α for high performance (8-10)
Correctness	8.71 (1.15)	0.85
Relevance	9.99 (0.10)	1.00
Clarity	9.33 (0.68)	0.95

a All results were generated using the 2025 system deployment. Three trained raters evaluated chatbot responses to food image-prompt pairs using 10-point Likert scales across 3 dimensions: correctness, relevance, and clarity. Mean scores and SDs are reported. Krippendorff α was calculated to assess interrater reliability for high-performance ratings (scores 8‐10).

1. Correctness (mean 8.71, SD 1.15): correctness ratings primarily reflect the accuracy of core elements, such as the identification of main ingredients and the alignment of the recommended cooking approach with the image content and user query. Chatbot responses were largely accurate, though minor discrepancies occurred with visually similar food items (eg, distinguishing arugula from green lettuce) or low-quality images.
2. Relevance (mean 9.99, SD 0.1): chatbot-generated responses appropriately addressed user queries, although context-specific prompts occasionally received generalized answers.
3. Clarity (mean 9.33, SD 0.68): responses were well-structured and aligned with training formats. However, output truncation due to token limitations occasionally resulted in incomplete responses.

A follow-up error analysis examined the nature of inaccuracies in chatbot-generated responses. Overall, 12% of responses exhibited ambiguity (eg, vague ingredient references or unclear procedural steps) and 56% contained hallucinations. An additional 4% of responses exhibited both ambiguity and hallucination, whereas 36% showed no detectable errors. In this analysis, hallucinations were defined as the inclusion of unsupported or inferred details, including commonly used ingredients and seasonings that were not visible in the input image but were mentioned in the responses. Importantly, hallucinations were coded even when the primary food identification and overall recipe logic were otherwise accurate. As a result, many responses that received high correctness scores in the human evaluation were still classified as containing hallucinations, reflecting that correctness and hallucination capture different aspects of response quality.

## Discussion

### Principal Findings

This study introduced the function and architecture of Purrfessor, a fine-tuned multimodal LLaVA chatbot designed for personalized dietary guidance through image-based and conversational interactions. Using a mixed methods evaluation framework that combined GPT-based recipe quality assessment, reference-free automated metrics, and human validation, we systematically examined the effects of domain-specific fine-tuning on food recognition and recipe generation tasks.

Across evaluations, Purrfessor consistently outperformed its raw LLaVA base model on all 7 GPT-4 rated recipe quality dimensions, with the largest gains observed in clarity, consistency, and completeness. Ingredient recognition under naturalistic prompting achieved a high average cosine similarity (0.90), indicating robust semantic alignment between detected foods and user-facing descriptions even when prompts did not explicitly request exhaustive food identification. These findings suggest that domain-specific fine-tuning can meaningfully enhance multimodal semantic grounding and response quality in dietary contexts.

At the same time, the evaluation results reveal important distinctions between different dimensions of system performance. High scores on clarity reflect the model’s strength in generating fluent, well-structured, and easily interpretable natural language responses. In contrast, the lower automated recipe coherence score highlights occasional limitations in maintaining correct temporal or logical sequencing of cooking steps. This divergence underscores that linguistic fluency and procedural reasoning represent related but distinct capabilities, and that strong natural language generation does not necessarily guarantee fully executable or logically optimal cooking procedures. Together, these findings illustrate the value of multidimensional evaluation frameworks for multimodal health AI systems, as different metrics capture complementary aspects of usability, correctness, and practical reasoning.

### Comparison to Prior Work

These findings build on and extend existing research in health chatbot design and multimodal AI. While previous research has established that LLMs can generate health-related responses with contextual relevance [1], relatively few have investigated the capabilities of integrated vision-language systems for food recognition and personalized nutrition guidance. Findings demonstrate that such systems, when fine-tuned with domain-specific data, can achieve high levels of semantic alignment in ingredient identification and generate user-aligned recipe recommendations.

Beyond usability, Purrfessor could enhance nutrition and meal planning among laypeople who may lack familiarity with digital environments. By leveraging natural language and image-based interactions, Purrfessor enables users to ask questions easily and receive prompt, understandable responses grounded in USDA-supported nutritional data, lowering the intimidation often associated with complex health apps or dense databases. Purrfessor can help lay users expand their nutrition knowledge by explaining the benefits of various food choices and suggesting recipes tailored to individual needs. For individuals with limited food resources, Purrfessor can offer practical suggestions for affordable alternatives that maintain essential nutrient intake, supporting healthier dietary practices in constrained contexts. Additionally, the chatbot’s friendly, anthropomorphic avatar design may support nutrition education among younger users, fostering healthy eating habits through daily engagement and gentle nudges toward better choices. Overall, this study reinforces the emerging view that multimodal, anthropomorphically designed chatbots can function not only as information tools but as relational companions in digital health contexts.

Meanwhile, recent work has increasingly adopted LLMs such as GPT-4 as evaluators for open-ended generation tasks, demonstrating strong alignment with human judgments on dimensions including clarity, relevance, and safety [35]. However, prior studies have also cautioned that LLM-as-a-Judge frameworks may overestimate performance when the evaluator shares training distributions or representational priors with the evaluated system. Consistent with this literature, this study treats GPT-based evaluation as an upper-bound estimate and complements it with reference-free, rule-based metrics that emphasize procedural correctness and executability. This combined approach highlights both the strengths and limitations of current LLM-based evaluation paradigms in multimodal health AI.

### Limitations

Despite the promising findings of this study, several limitations should be acknowledged. First, the chatbot’s context awareness was limited to session-level memory. While the system supports multiturn interactions and can maintain conversational context within an active session, it does not retain conversation history once the session is closed or the interface is reloaded. As a result, contextual continuity across separate user visits is not preserved. While findings highlight the importance of persistent memory for improving long-term personalization and conversational coherence, this study did not evaluate specific implementation strategies for cross-session memory retention.

Second, although the core chatbot model is based on LLaVA-v1.6-13B, GPT-4o was used to assist with structured training data generation, and a GPT-4–based model was used as an evaluator under the G-Eval (“LLM-as-a-Judge”) framework. While training data were subsequently reviewed and edited by human annotators and the evaluation model was not used for training, this model-family overlap introduces a potential circular validation bias that may inflate performance estimates. Accordingly, results derived from G-Eval should be interpreted as upper-bound estimates rather than fully independent validations.

Third, the relevance dimension showed a near-ceiling effect (mean 9.99, SD 0.10) in human scoring, likely reflecting the design limitations of the human evaluation rubric. Relevance was defined primarily in terms of task-level appropriateness, whether the response directly addressed the user’s question, rather than the degree of contextual specificity or personalization. Consequently, responses that were occasionally described qualitatively as generalized could still receive high relevance scores if they adequately addressed the prompt.

Fourth, although the study distinguishes hallucinations based on visual or presentational salience, hallucinations involving nutritionally or medically relevant ingredients (eg, salt, oil, and sugar) may pose material risks for users with specific dietary restrictions or chronic health conditions. While such ingredients may be visually inconspicuous, their presence or absence can have meaningful health implications in certain contexts. The current system does not implement ingredient-level verification or medical rule enforcement, as introducing strict constraints or external validation layers may reduce the flexibility and creativity that are central to recipe generation and user engagement.

Additionally, while real-world food images were used to enhance ecological realism at the input level, the evaluation relied on synthetically generated prompts for standardization. Such prompts may not fully capture the variability and noise of unconstrained human interactions, and thus, the findings primarily reflect model performance under structured task conditions.

Finally, although the fine-tuned Purrfessor model is compared against its raw LLaVA base to isolate the effects of domain-specific fine-tuning, this evaluation does not establish comparative performance against other state-of-the-art dietary assistants or text-only LLMs provided with structured ingredient inputs. Thus, the results should be interpreted as evidence of within-architecture improvement rather than global model superiority.

### Future Work

Future research may explore ways to support continuity across user sessions, allowing the system to maintain context over time while carefully addressing privacy, data governance, and efficiency considerations. Longitudinal studies of sustained use would help clarify how conversational continuity influences personalization, user experience, and dietary decision support. In addition, future development may consider incorporating clearer safeguards around ingredient accuracy, particularly for individuals with dietary restrictions or chronic health conditions, where small inaccuracies could have meaningful implications.

Methodological improvements are also needed to strengthen evaluation rigor. Future studies may include independent evaluation approaches that do not rely on LLMs, as well as greater involvement of domain experts in assessing output quality. Refining human evaluation instruments to better capture contextual depth and personalization could further distinguish between responses that simply address a prompt and those that demonstrate more adaptive, situation-specific reasoning.

Finally, future research should assess system performance under more naturalistic conditions. Collecting and analyzing organically generated user prompts would provide insight into how the system performs amid the variability and unpredictability of real-world interactions. Broader comparisons with other multimodal and text-based systems would also help determine whether the observed improvements reflect model-specific tuning effects or more general advances in multimodal dietary guidance.

### Conclusions

This study presented the design and evaluation of Purrfessor, a fine-tuned multimodal LLaVA-based chatbot developed to support multimodal dietary guidance. Using a mixed methods evaluation framework that combined simulation testing, automated metrics, and human scoring, we assessed the system performance in ingredient recognition and recipe generation quality.

Compared with the base LLaVA model, the fine-tuned Purrfessor model demonstrated improved semantic alignment in ingredient recognition and achieved higher scores across all 7 GPT-4.1–rated recipe quality dimensions, including correctness, clarity, completeness, consistency, practicality, and safety. Automated structured metrics further identified strengths in ingredient coverage and step complexity, alongside limitations in procedural coherence and temperature and time specification. Human evaluations further showed high ratings for correctness, relevance, and clarity of chatbot responses, while error analysis highlighted the presence of hallucinations in secondary details, underscoring the need to interpret accuracy and safety as distinct dimensions of system performance.

These findings indicate that domain-specific fine-tuning of a multimodal vision-language model can improve performance on food-related reasoning tasks under controlled simulation conditions. The results provide empirical evidence regarding the measurable capabilities and limitations of multimodal dietary chatbots evaluated through mixed methods assessment.

## Supplementary material

10.2196/74111 Multimedia Appendix 1 Human scoring example.

10.2196/74111 Multimedia Appendix 2 Human scoring criteria.

## Abbreviations

AI: artificial intelligence
API: application programming interface
GPU: Graphics Processing Unit
LLaVA: Large Language and Vision Assistant
LLM: large language model
LoRA: Low-Rank Adaptation
Q&A: question and answer
USDA: US Department of Agriculture
