# Supplementary material for: A Fine-Tuned Multimodal AI Chatbot for Dietary Health and Nutrition, Purrfessor: Development and Mixed Methods Evaluation
Source: JMIR AI. 2026 Apr 30;5:e74111. doi: 10.2196/74111 (PMC13132530; doi:10.2196/74111)
Supplement: Multimedia Appendix 1 [file ai-v5-e74111-s001.docx]

**Appendix 1.** Human Scoring Example

| Q&A example for image (507_65fc368e13afe3248977718dc0a471cc.jpg) 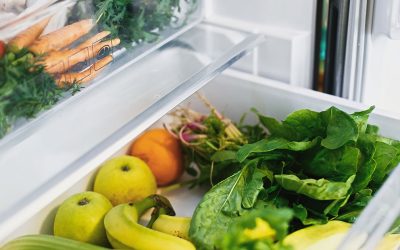 |
| --- |
| ****Question:**** What can I prepare using the fresh ingredients I see in my fridge?  ****Response from Purrfessor:****  1. **Greeting**  Hello! You've got a wonderful selection of fresh ingredients. Let's whip up something tasty and nutritious!  2. **Ingredients Overview**  Here's what you have:  - **Leafy Greens:** High in vitamins A, C, and K, and a great source of fiber.  - **Apples:** Rich in fiber and vitamin C.  - **Carrots:** Packed with beta-carotene, fiber, and vitamin A.  - **Bananas:** A good source of potassium and vitamin B6.  - **Lettuce:** Low in calories and a good source of vitamins A and K.  3. **Suggested Recipes**  With these ingredients, you can try these healthy recipes:  - **Green Apple and Carrot Salad**  - **Banana and Spinach Smoothie**  4. **Cooking Guide**  **Green Apple and Carrot Salad**  **Ingredients:**  - 1 green apple, sliced  - 2 carrots, grated  - 1/4 cup walnuts, toasted  - 1/4 cup feta cheese, crumbled  - 1/4 cup honey  - 1/4 cup olive oil  - Salt and pepper to taste  **Steps:**  1. In a large bowl, combine the sliced green apple, grated carrots, and toasted walnuts.  2. In a small bowl, whisk together honey, olive oil, salt, and pepper to make the dressing.  3. Drizzle the dressing over the salad and toss gently to coat.  4. Top with crumbled feta cheese and serve immediately.  5. **Closing**  Enjoy your delicious and nutritious salad! If you need more recipe ideas or cooking tips, please feel free to ask. Happy cooking! |

**Human Scoring Results**

| Criteria | Definition | Evaluation | Score (1-10) | |
| --- | --- | --- | --- | --- |
| Correctness | Evaluates the accuracy of the image object detection by comparing the detected food items in the image with the provided text. | Moderate – The chatbot accurately identifies most of the visible items (leafy greens, apples, carrots, bananas). Lettuce is not clearly visible in the image. | | 9 |
| Relevance | Evaluates the pertinence of the chatbot's responses to the questions asked. | High – The response provides relevant recipe suggestions based on the identified ingredients, including a salad and smoothie that align with the contents of the fridge. | | 10 |
| Clarity | Measures the ease with which the chatbot’s responses can be understood by users. | High – The response is clear and structured, with organized steps and simple instructions. The response is easily understandable for users looking for quick meal ideas. | | 9 |
