# Supplementary material for: A Fine-Tuned Multimodal AI Chatbot for Dietary Health and Nutrition, Purrfessor: Development and Mixed Methods Evaluation
Source: JMIR AI. 2026 Apr 30;5:e74111. doi: 10.2196/74111 (PMC13132530; doi:10.2196/74111)
Supplement: Multimedia Appendix 2 [file ai-v5-e74111-s002.docx]

**Appendix 2.** Human Scoring Criteria

| **Criteria** | **Definition** | **Score (1-10)** | **Scoring Degree** |
| --- | --- | --- | --- |
| Correctness | Evaluates the accuracy of the image object detection by the provided text in **Ingredients Overview**. | Deduct 1 point for each incorrectly identified. | High (8-10)  Medium (4-7)  Low (1-3) |
| Relevance | Evaluates the degree to which the chatbot's responses appropriately address the user’s question. | Holistic score based on response appropriateness. | High (8-10): Directly answers the question  Medium (4-7): Partially relevant or overly generic  Low (1-3): Off-topic or unresponsive |
| Clarity | Measures the ease with which the chatbot’s responses can be understood by users. | Holistic score based on response appropriateness. | High (8-10): Clear, concise, and well-structured  Medium (4-7): Somewhat clear but may contain ambiguities  Low (1-3): Difficult to understand or poorly structured |
